# Identification of DNA sequence variation in *Campylobacter jejuni *strains associated with the Guillain-Barré syndrome by high-throughput AFLP analysis

**DOI:** 10.1186/1471-2180-6-32

**Published:** 2006-04-04

**Authors:** Peggy CR Godschalk, Mathijs P Bergman, Raymond FJ Gorkink, Guus Simons, Nicole van den Braak, Albert J Lastovica, Hubert P Endtz, Henri A Verbrugh, Alex van Belkum

**Affiliations:** 1 Department of Medical Microbiology & Infectious Diseases, Erasmus MC – University Medical Center Rotterdam, Dr. Molewaterplein 40, 3015 GD Rotterdam, The Netherlands; 2 Department of Microbial Genomics, Keygene NV, Agro Businesspark 90, 6708 PW Wageningen, The Netherlands; 3 Pathofinder BV, Canisius Wilhelmina Hospital, Weg door Jonkerbos 100, 6532 SZ, Nijmegen, The Netherlands; 4 Department of Clinical Laboratory Sciences, Division of Microbiology, and IIDMM, University of Cape Town, Anzio Road, Observatory 7925, Cape Town, South Africa

## Abstract

**Background:**

*Campylobacter jejuni *is the predominant cause of antecedent infection in post-infectious neuropathies such as the Guillain-Barré (GBS) and Miller Fisher syndromes (MFS). GBS and MFS are probably induced by molecular mimicry between human gangliosides and bacterial lipo-oligosaccharides (LOS). This study describes a new *C. jejuni*-specific high-throughput AFLP (htAFLP) approach for detection and identification of DNA polymorphism, in general, and of putative GBS/MFS-markers, in particular.

**Results:**

We compared 6 different isolates of the "genome strain" NCTC 11168 obtained from different laboratories. HtAFLP analysis generated approximately 3000 markers per stain, 19 of which were polymorphic. The DNA polymorphisms could not be confirmed by PCR-RFLP analysis, suggesting a baseline level of 0.6% AFLP artefacts. Comparison of NCTC 11168 with 4 GBS-associated strains revealed 23 potentially GBS-specific markers, 17 of which were identified by DNA sequencing. A collection of 27 GBS/MFS-associated and 17 enteritis control strains was analyzed with PCR-RFLP tests based on 11 of these markers. We identified 3 markers, located in the LOS biosynthesis genes cj1136, cj1138 and cj1139c, that were significantly associated with GBS (P = 0.024, P = 0.047 and P < 0.001, respectively). HtAFLP analysis of 13 highly clonal South African GBS/MFS-associated and enteritis control strains did not reveal GBS-specific markers.

**Conclusion:**

This study shows that bacterial GBS markers are limited in number and located in the LOS biosynthesis genes, which corroborates the current consensus that LOS mimicry may be the prime etiologic determinant of GBS. Furthermore, our results demonstrate that htAFLP, with its high reproducibility and resolution, is an effective technique for the detection and subsequent identification of putative bacterial disease markers.

## Background

The Guillain-Barré syndrome (GBS) is the most frequent form of acute immune-mediated neuropathy. The Miller Fisher syndrome (MFS) is a variant of GBS, affecting mainly the eye muscles [1]. A respiratory or gastro-intestinal infection preceding the neurological symptoms is reported by nearly two-thirds of all patients [2]. The most frequently identified infectious agent is *Campylobacter jejuni*, which is also the predominant cause of bacterial diarrhoea worldwide [3,4]. The neuropathy is probably induced by molecular mimicry between gangliosides in nerve tissue and lipo-oligosaccharides (LOS) on the *Campylobacter *cell surface [5]. This structural resemblance leads to a cross-reactive immune response causing neurological damage. Biochemical and serological studies have revealed that many *C. jejuni *strains express ganglioside-like structures in their LOS [6]. However, not all strains expressing ganglioside mimics induce GBS. It is estimated that only 1 in every 1000–3000 *C. jejuni *infections is followed by GBS [7,8], which suggests that additional bacterial determinants and/or host-related factors are important as well.

Many researchers have studied collections of GBS-associated and control "enteritis-only" strains in search of GBS-specific microbial features. Several reports describe an overrepresentation of specific Penner (heat stable, HS) serotypes among GBS-associated strains from certain geographical areas [9,10]. The HS:19 and HS:41 serotypes are the predominant serotypes preceding GBS in Japan and South Africa, respectively [9,10]. Because HS:19 and HS:41 strains represent a clonal population [11,12], the observed overrepresentation of these serotypes does not imply that the determinant of the Penner serotyping system, the capsular polysaccharide, is involved in the pathogenesis of GBS [13]. In addition, this phenomenon is not seen in other regions, where GBS-associated strains are genetically heterogeneous [14]. Various molecular typing techniques have been used in search of GBS-specific features in *C. jejuni*, such as *flaA*-PCR-restriction fragment length polymorphism (RFLP), pulsed field gel electrophoresis (PFGE), randomly amplified polymorphic DNA (RAPD) analysis, ribotyping, amplified fragment length polymorphism (AFLP) analysis and multi locus sequence typing (MLST), but none of these have identified GBS-specific markers [14-17]. Very recently, Leonard et al. also failed to detect GBS-specific features by the use of an open reading frame (ORF)-specific *C. jejuni *DNA microarray [18]. However, this array was based on the genome sequence of strain NCTC 11168 and ORFs that are not present in this strain will not be detected. In addition, possible GBS-factors other than those relating to presence or absence of certain genes will not be detected using this approach. Based on the molecular mimicry hypothesis, other researchers focused on genes involved in LOS biosynthesis and found significant associations with GBS [19-22]. However, these associations are not absolute and the question remains whether other GBS-specific microbial factors, either LOS-related or not, may exist.

Comparative genomics technology facilitates genetic marker identification but not all methods may be equally suited for high-throughput marker searches. Molecular typing techniques for *Campylobacter *strains differ in sensitivity and the overall coverage of genome regions screened. For the detection of specific disease markers it is desirable to use a technique that screens diversity in the overall genome with a very high resolution. MLST and *flaA *PCR-RFLP analyze restricted parts of the genome and are not suitable for the detection of additional GBS-markers [23,24]. PFGE is based on digestion of genomic DNA with a rare cutting restriction enzyme and only large insertions or deletions and mutations in the restriction sites will be detected [14]. PFGE patterns normally display between 10 and 20 fragments, which covers 120 nucleotides when a six nucleotide restriction enzyme recognition sequence is involved. AFLP analysis is considerably more sensitive than PFGE for the detection of DNA sequence polymorphism. In a conventional AFLP analysis, the use of two restriction enzymes and a primer pair with 1 or 2 selective nucleotides leads to a DNA fingerprint pattern consisting of approximately 50–80 fragments per strain. This approach physically covers in the order of 600–1000 nucleotides of the total genome for sequence polymorphism [25]. Even the use of two restriction enzymes in PFGE will not make up for the difference observed under a single AFLP reaction. As indicated above, DNA microarrays cover the full genome but will only detect differences in the presence of known genes. Recently, we described a new high throughput AFLP (htAFLP) approach for the identification of DNA polymorphism in *Mycobacterium tuberculosis *[26]. This method has the capacity to detect mutations in more than 30,000 nucleotides scattered throughout the genome, depending on the number of restriction enzymes and primer pairs used. The choice of primers and restriction enzymes is crucial and selection of these requires close attention. A wrong choice may lead to crowding of amplified fragments, caused by limiting (limited?) resolution of the gelsystem. Correct, computer-mediated comparison of AFLP fingerprints may then be compromised. However, especially the enhanced resolution makes htAFLP an excellent candidate technique for the detection of potential disease-associated markers.

The main objective of the current study was to search an elaborate collection of C. jejuni stains isolated from GBS patients for genetic markers associated with bacterial neuropathogenicity. To this aim, we developed htAFLP for *C. jejuni*. We analyzed six isolates of strain NCTC 11168, the "genome" strain, obtained from different laboratories, with the aim to detect base-level polymorphism introduced by sub-culturing or storage. In search of potential GBS-specific markers, we compared the NCTC 11168 AFLP patterns with those of four GBS-associated strains. In addition, we analyzed a collection of highly clonal GBS-associated and control strains from South Africa. Potential GBS-specific htAFLP markers were further identified by DNA sequencing and PCR-RFLP tests were developed. These PCR-RFLP tests were used to screen a larger collection of GBS-associated and control strains for confirmation of the potential GBS markers.

## Results

### Detection and identification of DNA sequence polymorphism in NCTC 11168 strains of diverse origin

HtAFLP analysis of *C. jejuni *was performed with one enzyme combination. Genomic DNA was digested with MboI and DdeI and the restricted DNA was amplified by using all 64 possible combinations of +1/+2 selective primer pairs. This resulted in the generation of approximately 3000 fragments per strain. The average fragment size was 243 basepairs (bp), ranging from 46 to 613 bp. Comparative htAFLP analysis of the six NCTC 11168 isolates revealed 19 polymorphic fragments. After excision from the gel, these fragments were amplified and the DNA sequences were determined. BLAST analysis of the DNA sequences resulted in the identification of 13/19 polymorphisms, which were spread throughout the genome (Table 1). For the other polymorphic fragments, repeated amplification and sequencing failed to generate DNA sequences of sufficient quality for BLAST analysis.

### Validation of NCTC 11168 polymorphism with a PCR-RFLP approach

To verify whether the htAFLP-polymorphic fragments represent true DNA sequence polymorphism, we analyzed the six NCTC 11168 isolates by PCR-RFLP analysis. An AFLP polymorphism that is based on mutations in the restriction site will result in a polymorphic RFLP pattern, whereas insertions or deletions in the AFLP fragment will result in size differences between the PCR products. Based on the BLAST hit sequences (Table 1), PCR tests for amplifying twelve marker fragments and their flanking regions were developed. Fragments of correct size were produced with all primer sets and for all isolates (results not shown). Next, PCR products of the six NCTC 11168 strains were digested in separate reactions with the AFLP restriction enzymes (results not shown). None of the digests showed polymorphic RFLP patterns. Thus, restriction site polymorphism or insertions/deletions could not be confirmed as cause of the observed AFLP polymorphisms. Nine out of twelve (75%) digests with *DdeI *and six of twelve (50%) digests with *MboI *resulted in RFLP patterns as expected based on the NCTC 11168 DNA sequence. An AFLP polymorphism can also be the result of a mutation in the nucleotides complementary to the selective primer nucleotides. Because all 64 possible combinations of the +1/+2 primer pairs were used in this htAFLP, such a mutation would be expected to result in an additional polymorphism, with the same fragment length and localization on the genome, in the AFLP pattern generated with a different primer pair. We did not detect such complementary polymorphisms in the NCTC 11168 comparison. In conclusion, the polymorphic AFLP bands observed in the NCTC 11168 comparison, representing approximately 0.6% of all bands, probably represent the low "background noise" of the htAFLP technique.

### Comparison of NCTC 11168 with GBS-associated strains for the detection and identification of potential markers for GBS

**Figure 1:**
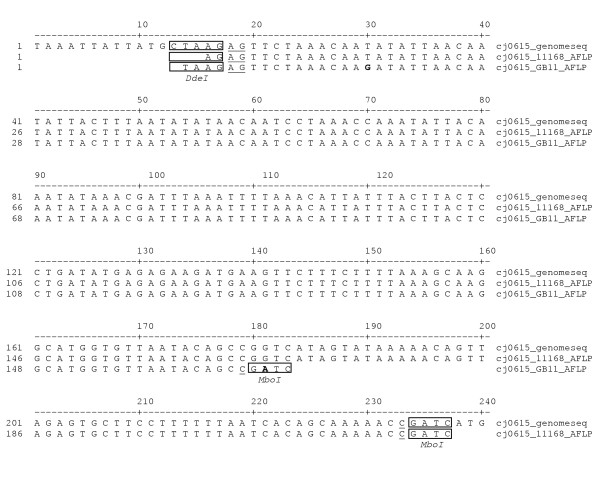
**The basis of the AFLP polymorphism in the complementary markers 5 and 6 **Marker 5 represents a fragment with a length of 198 bp that was present in the ht AFLP GBS strains, whereas marker 6, a fragment of 253 bp, was only present in the NCTC 11168 isolates. DNA sequence analysis of the GB11 and NCTC 11168 AFLP fragments and subsequent BLAST searches showed that both markers were located in gene cj0615, encoding a possible periplasmic protein. Furthermore, DNA sequence analysis revealed that a point mutation in the GBS strains had resulted in an additional MboI restriction site, resulting the amplification of a shorter AFLP fragment in the GBS strains. Because the selective nucleotide flanking the *Mbo*I restriction site was identical for the GB11 and NCTC 11168 fragment, the GBS fragments were amplified with the same primerpair as the NCTC 11168 fragments. DNA sequences of markers 5 (cj0615_GB11_AFLP) and 6 (cj0615_NCTC 11168_AFLP) are given, as well as the NCTC 11168 genome sequence of the same area (cj0615_genomeseq). The AFLP restriction sites are indicated with boxes, the selective nucleotides are underscored and the point mutation is indicated in bold.

For the detection of putative markers for the Guillain-Barré syndrome, we compared the NCTC 11168 isolates with strain GB11, which was isolated from a GBS patient. Strain NCTC 11168 was originally isolated from a patient with gastroenteritis without neurological symptoms. It had previously been shown that GB11 and NCTC 11168 are genetically closely related [14,15,27]. Because of this relatedness, these strains are very suitable for the detection of potential GBS-specific markers. HtAFLP analysis of NCTC 11168 and GB11 generated 241 putative GBS markers. Overall, 156 of 241 markers could be successfully identified with DNA sequencing and BLAST analysis. A proportion of the marker fragments that were excised from the gel could not be reliably reamplified and were excluded from the analysis. Although BLAST searches were conducted against all DNA sequences in the Pubmed database, the most significant homology for all AFLP DNA sequences was with *C. jejuni *DNA sequences. To further reduce this excessive number of putative GBS markers, we analyzed three additional GBS-associated strains, not related to the NCTC 11168 and GB11 strains (Cura7, Cura276 and 260.94; See Additional file 1). This reduced the number of successfully sequenced putative GBS markers to 17 (Table 2). Three of these markers were located in the LOS biosynthesis gene locus. Other genes encoded a putative periplasmic protein and proteins involved in signal transduction, metabolism, transport, binding, amino acid biosynthesis, fatty acid biosynthesis and DNA replication. Three genes were of unknown function. Markers 5 and 6 displayed distinct restriction site polymorphism concordant with the AFLP polymorphism. These markers contained largely overlapping DNA sequences and showed a complementary pattern of presence and absence in the GBS-associated and control strains (Table 2). Comparison of the DNA sequences revealed that these markers were based on one DNA polymorphism: the presence of an additional restriction site in the GBS strains due to a point mutation (Figure 1).

### Screening of a large strain collection for potential GBS markers by PCR-RFLP analysis

After htAFLP analysis of five strains to identify potential GBS markers, we developed PCR-RFLP tests to screen a large collection of 27 GBS/MFS-associated and 17 control enteritis strains for the presence of these markers (for a survey of these strains see Additional file 1). The strains used in the htAFLP analysis were also included in the PCR-RFLP analysis. One randomly selected NCTC 11168 isolate was included. Based on the BLAST hit sequences (Table 2), PCR tests for amplifying 11/17 marker fragments and their flanking regions were developed (See Additional file 2). Because markers 5 and 6 represented the same DNA polymorphism, they were included in one PCR test. Fragments of correct, expected size were produced with all primer sets. For 5/11 markers a PCR product was absent in a variable proportion of strains (Table 3), probably due to primer site sequence heterogeneity. For example, we have observed previously that gene *cj1136*, part of the LOS biosynthesis gene locus and containing marker 7, shows a large degree of DNA sequence heterogeneity between strains (P. Godschalk, unpublished results). This leads to primer mismatches and absence of PCR products in a proportion of strains. In 2/10 PCR tests (markers 7 and 8), the htAFLP GBS-associated strains could be distinguished from strain NCTC 11168 through the pattern of presence and absence of PCR products. PCR products for marker 7 were absent in the GBS-associated strains used in the htAFLP and present in NCTC 11168, which seemed to be in contrast with the observation that the original AFLP fragment of marker 7 was present in the GBS strains and absent in NCTC 11168. However, this apparent inconsistency can be explained by the fact that the primer sequences of the PCR test were based on the NCTC 11168 DNA sequence. For marker 7, a PCR product was seen significantly more frequently in control enteritis strains (5/27 (18.5%) GBS/MFS strains versus 9/17 (52.9%) control enteritis strains, *P *= 0.024). Next, we subjected the PCR products to a combined digestion with the AFLP restriction enzymes (Table 3). In 4/10 PCR tests (markers 3, 5/6, 9 and 13), the RFLP types were concordant with the AFLP analysis i.e. the htAFLP GBS-associated strains shared the same RFLP type whereas NCTC 11168 displayed a different RFLP type. In 3/10 PCR tests (markers 11, 12 and 14), the htAFLP GBS-associated strains did not have identical RFLP types (and for marker 14 there was no PCR product in two GBS strains), but these RFLP types were also different from that of NCTC 11168. This is not necessarily in contrast with the htAFLP results, because different RFLP types among the htAFLP GBS-associated strains may be due to heterogeneity in the flanking regions of the AFLP fragment. For marker 2, the RFLP types of the htAFLP strains were not concordant with the htAFLP polymorphism: the NCTC 11168 and GB11 RFLP types were the same. RFLP types 3 and 4 of marker 9, located in gene cj1139c, were only detected in GBS/MFS-associated strains (RFLP type 3 or 4 present in 15/27 (55.6 %) GBS/MFS-associated strains versus 0/17 (0%) enteritis strains, P <0.0001). Although a PCR product for marker 8, located in gene cj1138, was absent in the majority of strains, RFLP type 1 was more frequently found in enteritis strains (5/15 enteritis strains versus 1/27 GBS-associated strains, P = 0.047).

### Analysis of South-African GBS-associated and control HS:41 strains by htAFLP

**Figure 2:**
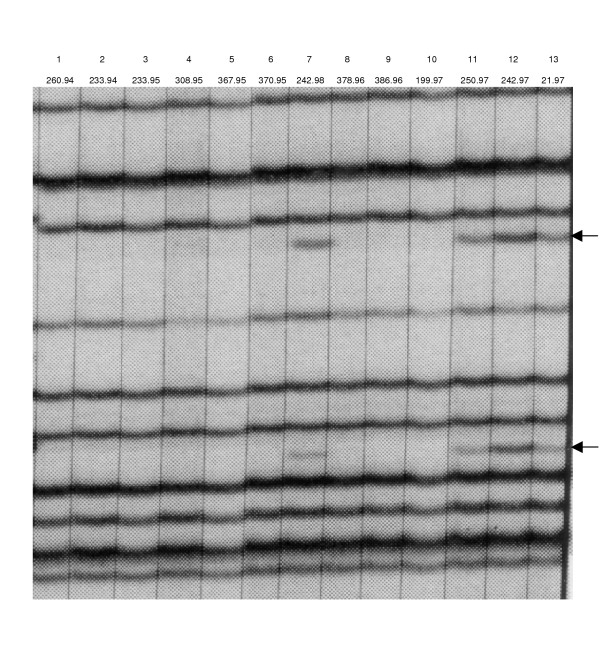
**Detail of the htAFLP pattern of the HS:41 strains **A fragment of the banding patterns of the South African HS:41 strains is shown. Lane 1–6 GBS-associated strains, lane 7 MFS-associated strain, lane 8–13 enteritis strains. Polymorphic bands representing plasmidal DNA sequences are indicated with arrows.

In South Africa, serotype HS:41 is over-represented among GBS-associated strains [10]. Previous studies have shown that HS:41 strains, both GBS-associated and controls, are highly clonal [12]. As expected, htAFLP of six GBS-associated, one MFS-associated and six control HS:41 strains generated very homogeneous banding patterns (results not shown). A total of forty-five AFLP polymorphisms were detected, but there were no GBS-specific markers. Interestingly, 28 AFLP polymorphisms displayed a similar pattern: fragments were present in the MFS-associated strain and two or three control enteritis strains but absent in the other strains (Figure 2). These fragments were excised and DNA sequences were determined. BLAST analysis of five DNA sequences revealed homologies with various bacterial plasmidal DNA sequences (results not shown).

## Discussion

This study describes a high-throughput AFLP approach for the detection and identification of DNA polymorphism and putative GBS-markers in *C. jejuni*. Previously, we showed that htAFLP is an excellent tool for assessing the population structure and the expansion of pathogenic clones in *Staphylococcus aureus *and for identification of genetic polymorphism in the clonal microorganism *Mycobacterium tuberculosis *[26,28]. The optimal enzyme and selective primer pair combinations are determined by *in silico *calculations using the whole genome DNA sequence of the target microorganism. The optimal number of AFLP fragments to be generated depends on the aim of the study. For the detection and identification of potential disease markers, such as GBS-specific markers in *C. jejuni *in the current study, it is desirable to screen the genome with high resolution. For this, the generation of a large number of AFLP fragments per strain is needed. Such high resolution AFLP approach limits the number of strains that can be analyzed, but this can be overcome by the subsequent analysis of a large number of strains by PCR-RFLP tests, translated from the potential markers as detected by the preceding htAFLP analysis. It is, of course, possible that a disease-specific marker is not detected by htAFLP because this approach does not result in 100% genome coverage, which can only be reached with whole genome sequencing. For *C. jejuni*, one enzyme combination (*Mbo*I and *Dde*I) and 64 different +1/+2 selective primer pair combinations physically covered approximately 30,000 nucleotides per strain, which represents approximately 2% of the genome.

To find out whether subculturing or storage of *C. jejuni *strains leads to the emergence of DNA polymorphism, we compared 6 isolates of the "genome" strain NCTC 11168 obtained from different laboratories worldwide by htAFLP analysis. The observed AFLP polymorphisms, approximately 0.6% of the fragments per strain, could not be confirmed by PCR-RFLP analysis. This indicates that the AFLP polymorphisms probably represent the low background noise of the htAFLP technique and that true DNA polymorphism could not be detected in the six NCTC 11168 isolates. The observed background noise equals a reproducibility of approximately 99.6%, which is still very high when compared to other genotyping techniques [29].

Recently, two groups described differences in virulence properties, i.e. the ability to colonise chickens, between different NCTC 11168 isolates that were not included in the current study [30,31]. Full transcriptional profiling revealed expression differences for several gene families in the NCTC 11168 strains. HtAFLP analysis of the two NCTC 11168 isolates that were studied by Carrillo et al. [30,31] failed to identify polymorphisms responsible for the difference in virulence properties (P. Godschalk and C. Szymanski, unpublished data). It has to be emphasized that by htAFLP still only a random proportion of the genome is screened. DNA sequence variation (such as single-nucleotide polymorphisms, SNPs) leading to biological differences may therefore not be detected.

In search of GBS-specific markers, we first compared the NCTC 11168 patterns with the AFLP patterns of the genetically related GBS-associated strain GB11. However, although NCTC 11168 was originally isolated from the faeces of a patient with gastroenteritis, we cannot exclude that NCTC 11168 can induce GBS if a patient with the right host susceptibility factors becomes infected with this strain. The only substantial but probably very important difference between NCTC 11168 and GB11 that has been found so far, is that the LOS biosynthesis gene locus strongly diverges between these strains, probably as result of a horizontal exchange event [32]. Comparison of NCTC 11168 with GB11 led to the detection of more than two hundred possible GBS markers, which was substantially higher than the expected background noise of 0.6%, underscoring the phylogenetic relevance of the polymorphisms. The number of possible GBS markers was reduced to 23 after adding three additional GBS-associated strains. For 17 markers, the location on the genome could be identified after DNA sequence analysis. A relatively large proportion of potential GBS markers (3/17;18%) was located in the LOS biosynthesis gene locus, whereas this locus only comprises 1% of the *C. jejuni *genome (1.64 Mbp). Although this may represent a true pathogenic association with GBS, it is also possible that htAFLP preferentially picked up the LOS locus because it is a highly polymorphic region. However, analysis of a larger *C. jejuni *strain collection by PCR-RFLP analysis showed that the three LOS-specific markers were indeed associated with GBS (marker 7, *P = *0.024; marker 8, *P = *0.047; marker 9, *P <*0.001). These findings are concordant with the proposed pathogenic mechanism of GBS and with previous reports that certain genes involved in LOS biosynthesis or specific nucleotide sequences within these genes occur more frequently in GBS-associated *C. jejuni *strains [19-22].

There are several possible explanations for the fact that we did not find molecular markers that are 100% specific for the Guillain-Barré syndrome. First, it is possible that truly GBS-specific features do not exist in *C. jejuni*. There is a broad variability in the severity and spectrum of clinical symptoms in GBS patients [33]. Different ganglioside mimicking structures and anti-ganglioside antibody specificities have been associated with certain clinical presentations [34-36], and therefore, *C. jejuni *markers may be associated with a subset of various disease entities. Because of this heterogeneity and the presumed importance of host-related factors, the existence of features in *C. jejuni *that are specific for GBS may be questionable. Second, a certain combination of multiple *C. jejuni *genes may be required for the induction of GBS. Detection of such combinations of markers ("polygenic markers") is extremely labour-intensive and cannot be achieved with the current approach. Finally, it is possible that htAFLP failed to detect GBS-specific markers because htAFLP does not accomplish 100% genome coverage.

One of the three additional GBS-associated strains mentioned above was from a collection of South-African HS:41 strains. In South Africa, the HS:41 serotype is over-represented among GBS-associated strains [10]. A certain feature of these strains may be responsible for their capacity to trigger GBS. HS:41 strains were found to be indistinguishable by previous genotyping studies, indicating that HS:41 strains form a genetically stable clone [12]. It is important to note that the enteritis-only HS:41 strains may have the same GBS-inducing capacity as the GBS-associated strains, because host-related factors are also crucial for developing GBS. We analyzed both GBS-associated and control enteritis-only HS:41 strains, as well as an MFS-associated isolate by htAFLP. Although we did not detect GBS-specific bands, we found that the MFS-associated isolate and half of the enteritis-only strains contained several additional fragments that appeared to be linked. DNA sequences of these fragments showed homologies with plasmidal DNA sequences, indicating that a subset of the HS:41 strains contained a plasmid. To our knowledge, the South African HS:41 strains we used in this study have never been analyzed for the presence of plasmids. Whether the presence of a plasmid is of importance for the virulence or neuropathogenic potential of HS:41 strains currently remains unknown, but seems unlikely based on the distribution of plasmidal DNA in the tested strains.

## Conclusion

Previous searches for *C. jejuni *markers for GBS-invoking potential were unsuccessful when performed with general genotyping techniques. Some studies that focussed at specific loci or sometimes even specific genes found potential, though not absolute, GBS markers within the LOS biosynthesis genes [19-22]. We have used a method, htAFLP, that detects sequence polymorphisms in a wide, non-gene dependent scale. Theoretically approximately 2% of the total genome is covered by this approach. However, we still conclude that bacterial GBS markers are not absolute, limited in number and located in the LOS biosynthesis gene locus. This corroborates the current consensus that LOS mimicry with human gangliosides may be the prime etiologic determinant of GBS. In addition to bacterial factors, host-related factors probably play an important role in the pathogenesis of GBS as well.

Furthermore, our results demonstrate that htAFLP, with its high reproducibility and resolution, is an adequate technique for the detection and subsequent identification of putative disease and epidemiological markers. Analysis of a limited number of strains in great detail by htAFLP and subsequent screening of a large collection of strains with simple PCR-RFLP tests combines high sensitivity with the possibility to screen large groups of strains. This allows for the identification of regions of genomic instability or variability. Finally, htAFLP does not require complete genome sequences and it is not influenced by the presence of sequences not present in the genome strain(s). As such, htAFLP is the second best option, after complete sequencing of the genome of multiple strains, for the unbiased detection of genome polymorphisms associated with pathogenicity or other features of bacterial isolates from diverse clinical and environmental origin.

## Methods

### Bacterial strains, culture conditions and DNA isolation

The *C. jejuni *strains used for htAFLP analysis are described in Additional file 1. We collected 6 isolates of the "genome" strain NCTC 11168 strains from different labs worldwide [37]. For the detection of potential GBS markers, we included four *C. jejuni *strains isolated from the diarrhoeal stools of GBS patients from different geographical areas (The Netherlands, Curaçao, South Africa). In addition, we analyzed a collection of 6 GBS-associated, 1 MFS-associated and 6 enteritis-only HS:41 strains isolated from South African patients [12]. After identification of potential GBS markers by htAFLP analysis of these strains, we screened a larger collection of 27 GBS/MFS-associated and 17 control strains isolated from enteritis patients with PCR-RFLP tests for the presence of these markers (See Additional file 1). *C. jejuni *strains were cultured for 24–48 hours on blood agar plates in a micro-aerobic atmosphere at 37°C. DNA was isolated using the Wizard Genomic DNA Purification Kit (Promega, Madison, WI).

### High-throughput AFLP

AFLP analysis was performed essentially as described by Vos et al. [38]. The optimal enzyme and primer combinations for *C. jejuni *were determined using the predictive software package REcomb [39]. Digestion with *Mbo*I and *Dde*I (Boehringer-Mannheim, Mannheim, Germany) was combined with the ligation of a specific linker oligonucleotide pair (*Mbo*I: 5'-CTCGTAGACTGCGTACC-3' and 5'-GATCGGTACGCAGTCTAC-3'; *Dde*I: 5'-GACGATGAGTCCTGAG-3' and 5'-TNACTCAGGACTCAT-3'). Subsequently, a non-selective pre-amplification was performed using the *Mbo*I primer (5'-GTAGACTGCGTACCGATC-3') and *Dde*I primer (5'-GACGATGAGTCCTGAGTNAG-3'). The selective amplifications were performed using different linker-specific primer combinations. The ^33^P-labeled *Mbo*I primer was extended with a single nucleotide (+1), whereas the *Dde*I primer was equipped with a 3' terminal dinucleotide (+2). These nucleotides probe sequence variation beyond that present in the restriction site itself. All 64 possible extension combinations were used. Radioactive labelling was used to enable isolation of DNA fragments from gels for post-AFLP sequencing analysis. Amplified material was analyzed on 50x30 cm slabgels and the amplimers were visualized using phosphor-imaging. Post-AFLP, gels were fixed, dried and stored at ambient temperature.

### Marker selection and identification

Marker bands were scored using the automated interpretation software package AFLP QuantarPro (Keygene NV, Wageningen, The Netherlands), resulting in a binary table scoring marker fragment absence (0) or presence (1). Polymorphic marker fragments were validated by visual inspection of the autoradiographs. Bands differing in signal intensity were not considered to be polymorphic. A potential marker for GBS was defined as an AFLP polymorphism that discerns the GBS-associated strains from the NCTC 11168 isolates. Potential GBS marker fragments can either be present or absent in GBS-associated strains as compared to NCTC 11168.

Relevant fragments were excised from the gels and re-amplified using their matching AFLP consensus primer set without restriction site-specific +1 and +2 extension sequences attached. The amplimers were subjected to DNA sequencing using a 96-well capillary sequencing machine (MegaBACE; Amersham). For fragment identification, the DNA sequences were subjected to BLASTn and BLASTx searches through the NCBI website [40]. BLAST results enable genomic localization and gene annotation for the polymorphic marker fragments.

### Development of PCR-RFLP tests

PCR-RFLP tests were developed to confirm polymorphism in the different NCTC 11168 isolates and to screen a collection of *C. jejuni *GBS/MFS-associated and control strains. PCR-RFLP tests could only be developed for the markers of which the localization on the *C. jejuni *genome was identified. Forward and reverse primers were designed (Primer Designer 4, Sci Ed Software, North Carolina) and synthesized, located approximately 50–200 bp upstream or downstream of the homologous region, respectively (Table 2). Because of the wide range of melting temperatures (Tm) of the primers and the sometimes considerable differences in Tm between primers within one PCR reaction, a touch-down PCR approach was applied. The program consisted of 15 cycles of 1 min 94°C, 1 min 70°C minus 1°C for each following cycle (lowest temperature 55°C), 1 min 72°C, followed by 25 cycles of 0.5 min 94°C, 1 min Tm – 5°C and 1 min at 72°C. Tm – 5°C represents the lowest melting temperature of the two primers used in the reaction minus 5°C. This resulted in the amplification of not only the AFLP fragment itself, but also of their flanking sequences. Next, 15 μl of each PCR product was subjected to a separate or combined digestion with the restriction enzymes (1 unit/reaction) used for the AFLP (*MboI *and *DdeI*). After overnight incubation at 37°C, the digests were analyzed on 2% agarose gels. The PCR-RFLP analysis will reveal whether or not the AFLP variability was due to variation in the restriction sites (different RFLP patterns) or to insertions or deletions within the AFLP fragment (size differences in PCR products). AFLP variation due to differences in the selective extension nucleotides of the AFLP primers will not be detected using this approach.

## Abbreviations

AFLP amplified fragment length polymorphism

GBS Guillain-Barré syndrome

HS heat stable

htAFLP high-throughput AFLP

LOS lipo-oligosaccharide

MFS Miller Fisher syndrome

MLST multi locus sequence typing

ORF open reading frame

PCR-RFLP polymerase chain reaction – restriction fragment length polymorphism

PFGE pulsed field gel electrophoresis

## Authors' contributions

PCRG analyzed and interpreted the data and drafted the manuscript. MPB designed and carried out the PCR-RFLP marker analysis. RFJG performed the htAFLP analysis and DNA sequencing. GS participated in the design of the study and htAFLP setup. NVDB collected the strains, performed DNA extractions and participated in the marker identification. AJL provided the South African *C. jejuni *strains and participated in the design of the study. HPE participated in the design of the study and writing of the manuscript. HAV participated in the design of the study. AVB conceived and coordinated the study and participated in writing of the manuscript. All authors critically read the manuscript and approved the final version.

## Supplementary Material

Additional file 1 *C. jejuni *strains used in this study Description of data: a summary of all strains used in this study is given in this table, with strain numbers, associated disease, origin of the strain and technique(s) that were used to analyse the strains in this study. Click here for file

Additional file 2 Primer sequences used in the PCR-RFLP analysis for the validation of potential GBS markers
Description of data: (none, title sufficiently describes data) Click here for file

## Figures and Tables

**Table 1:** Polymorphic htAFLP markers for NCTC11168 strains of diverse origin

	NCTC11168 strains^b^							11168 hit sequence^d^		
				
marker^a^	1	2	3	4	5	6	length^c^	Begin	end	gene and function
1	+	+	+	+	-	+	469	28753	28317	Cj0023, purB, probable adenylosuccinate lyase
2	-	-	+	-	-	+	252	67665	67858	Cj0046, probable transmembrane transport protein pseudogene
3	-	-	+	-	-	-	110	122004	122083	Cj0117, pfs, probable 5'-methylthioadenosine\S-adenosylhomocysteine nucleosidase
4	-	-	+	+	+	+	118	153984	154075	Cj0150c, probable aminotransferase
5	-	-	+	+	+	+	86	212438	212383	Cj0227, argD, probable acetylornithine aminotransferase
6	+	+	+	+	+	-	420	573124	572971	Cj0612c, cft, probable ferritin; Cj0613, pstS, possible periplasmic phosphate binding protein
7	-	-	+	+	+	+	49	589048	589393	Cj0629, possible lipoprotein; Highly similar to Cj1678; Cj1678, possible lipoprotein
8	-	-	+	+	+	+	382	788562	788468	Cj0840c, fbp, probable fructose-1,6-bisphosphatase
9	-	-	+	-	-	-	463	1047891	1048322	Cj1116c, ftsH, probable membrane bound zinc metallopeptidase
10	-	-	-	+	+	-	228	1227296	1227125	Cj1295, unknown
11	+	-	-	+	-	-	226	1227296	1227117	Cj1295, unknown
12	+	+	-	+	+	+	191	1270291	1270131	Cj1339c, flaA, flagellin A
13	-	-	+	-	+	+	126	1510286	1510381	Cj1580c, probable peptide ABC-transport system ATP-binding protein

^a^The individual markers have been given a numerical code. ^b^The strains are numbered according to Additional file 1. The plusses and minuses indicate the presence and absence, respectively, of the AFLP fragments. ^c^The fragment lengths, based on fragment position in the AFLP gels. ^d^The begin and the end positions in the NCTC 11168 genome are given for all BLAST hits, as well as the corresponding genes and their function.

**Table 2:** Putative GBS-markers detected by htAFLP

	strains^b^						11168 hit sequence^d^		
marker^a^	11168	GB11	cura 7	cura 276	O:41	length^c^	begin	end	gene and function
				
1	-	+	+	+	+	112	324441	324363	Cj0355c, probable two-component regulator
2	+	-	-	-	-	265	400499	400264	Cj0432c, murD, probable UDP-N-acetylmuramoylalanine – D-glutamate ligase
3	-	+	+	+	+	115	575807	575893	Cj0615, pstA, probable phosphate transport system permease protein
4	-	+	+	+	+	152	576061	576182	Cj0615, pstA, probable phosphate transport system permease protein
5	-	+	+	+	+	198	904760	904594	Cj0967, possible periplasmic protein
6	+	-	-	-	-	253	904817	904596	Cj0967, possible periplasmic protein
7	-	+	+	+	+	241	1070741	1070662	Cj1136, probable galactosyltransferase
8	+	-	-	-	-	138	1073190	1073082	Cj1138, probable galactosyltransferase
9	-	+	+	+	+	97	1074261	1074320	Cj1139c, probable galactosyltransferase
10	+	-	-	-	-	169	1090751	1090612	Cj1157, dnaX, probable DNA polymerase III subunit gamma
11	+	-	-	-	-	89	1092245	1092185	Cj1161c, probable cation-transporting ATPase
12	+	-	-	-	-	181	1236007	1236080	Cj1306c, unknown
13	+	-	-	-	-	137	1236406	1236298	Cj1306c, unknown, similar to Cj1310c, Cj1305c, Cj1342c, Cj0617/0618
14	+	-	-	-	-	438	1264182	1264586	Cj1336, unknown, identical to Cj1318 (except the C-term)
15	+	-	-	-	-	163	1328311	1328184	Cj1394, probable fumarate lyase; Cj1393, metC', probable cystathionine beta-lyase
16	+	-	-	-	-	203	1334951	1334779	Cj1400c, fabI, probable enoyl- [acyl-carrier-protein] reductase [NADH]
17	+	-	-	-	-	102			Cj1101, probable ATP-dependent DNA helicase

^a^The individual markers have been given a numerical code. ^b^The plusses and minuses indicate the presence and absence, respectively, of the AFLP fragments. ^c^The estimated fragment lengths on the AFLP gels are given. ^d^The begin and the end positions in the NCTC 11168 genome are given for all BLAST hits, as well as the corresponding genes and their function. Note that markers 5 and 6 are largely overlapping and have a complementary pattern of presence and absence (see also Figure 1).

**Table 3:** Results of the PCR-RFLP analysis for 11 potential GBS markers identified by htAFLP analysis

		marker nr (genomic localization)^b^									
		
strain^a^	disease	2 (cj0432)	3 (cj0615)	5,6 (cj0967)	7 (cj1136)	8 (cj1138)	9 (cj1139c)	11 (cj1161c)	12 (cj1306)	13 (cj1306)	14 (cj1336)
GB1	GBS	1	1	1	1	1	0	1	2	2	1
GB2	GBS	2	2	2	-	-	1	2	4	3	-
GB3	GBS	2	2	2	-	-	1	2	3	3	-
GB4	GBS	1	2	2	-	-	2	2	1	1	-
GB5	GBS	1	2	1	-	-	3	0	5	4	-
MF6	MFS	2	2	2	-	-	4	2	6	0	-
MF7	MFS	1	2	1	-	-	3	1	5	4	-
MF8	MFS	1	2	2	-	-	0	2	6	1	-
**GB11**	GBS	1	2	2	-	-	4	2	7	5	-
GB13	GBS	1	2	1	1	0	0	2	5	1	-
GB15	GBS	1	1	3	1	-	0	2	5	4	2
GB16	GBS	1	1	1	-	-	3	2	1	1	-
GB17	GBS	1	2	1	-	0	0	1	5	4	-
GB18	GBS	2	2	2	-	-	4	2	4	6	-
GB19	GBS	1	1	1	-	-	5	2	3	7	-
MF20	MFS	1	2	3	-	-	5	1	5	8	-
GB21	GBS	2	2	2	-	-	4	2	0	5	3
GB23	GBS	1	2	1	-	-	4	0	1	1	4
GB24	GBS	2	1	2	-	-	-	1	1	4	-
GB25	GBS	1	2	-	-	-	3	1	5	8	-
GB26	GBS	1	1	1	-	-	3	1	5	1	-
GB29	GBS	1	2	1	1	-	-	1	10	10	3
GB30	GBS	3	1	3	1	-	3	1	1	1	5
**cura 7**	GBS	2	2	2	-	-	4	2	8	5	3
cura 69	GBS	2	2	2	-	-	4	2	3	9	-
**cura 276**	GBS	2	2	2	-	-	4	2	8	5	3
**260.94**	GBS	2	2	2	-	-	4	4	9	5	-
E97-0737	enteritis	1	2	1	1	nd	0	1	2	1	-
E97-0747	enteritis	1	1	1	1	nd	0	2	5	10	5
E97-0796	enteritis	1	2	1	1	-	0	1	5	4	6
E97-0873	enteritis	2	3	2	-	-	0	2	5	nd	4
E97-0903	enteritis	1	2	1	1	-	2	1	11	8	-
E97-0921	enteritis	1	2	3	-	-	6	0	11	6	-
E97-0974	enteritis	1	2	1	1	1	0	nd	5	1	4
E97-0980	enteritis	1	1	1	-	-	5	1	5	1	-
E97-0998	enteritis	2	2	2	-	-	7	2	11	nd	-
E97-1013	enteritis	1	1	3	-	-	5	1	5	10	-
E98-623	enteritis	2	2	2	-	-	2	2	5	nd	4
E98-624	enteritis	2	2	2	1	-	2	2	2	nd	7
E98-682	enteritis	1	1	1	1	1	0	1	2	10	-
E98-706	enteritis	1	1	3	-	-	0	2	1	4	-
E98-1033	enteritis	1	2	3	-	-	1	1	1	1	-
E98-1087	enteritis	1	2	1	1	1	0	1	1	4	-
**NCTC 11168-1**	enteritis	1	1	1	1	1	0	1	5	1	4

^a^Strains that were used in the htAFLP are indicated in bold. ^b^The marker numbers correspond with the marker numbers displayed in Table 3. For each marker, the different RFLP types are indicated with numbers. 0 = single band (no restriction), – = no PCR product, nd = not determined.
