# Supplementary material for: Identification of DNA sequence variation in Campylobacter jejuni strains associated with the Guillain-Barré syndrome by high-throughput AFLP analysis
Source: BMC Microbiol. 2006 Apr 4;6:32. doi: 10.1186/1471-2180-6-32 (PMC1513382; doi:10.1186/1471-2180-6-32)
Supplement: Additional file 1 — C. jejuni strains used in this study Description of data: a summary of all strains used in this study is given in this table, with strain numbers, associated disease, origin of the strain and technique(s) that were used to analyse the strains in this study. [file 1471-2180-6-32-S1.doc]

## Additional file 1 - *C. jejuni* strains used in this study

-----------------------------------------------------------------------------------------------------------------------------------------------------------------

strain associated disease origin used for

-----------------------------------------------------------------------------------------------------------------------------------------------------------------

NCTC 11168-1 enteritis ID-DLO, Lelystad, The Netherlands htAFLP, PCR-RFLP

(obtained from **NCTC**, UK)

NCTC 11168-2 enteritis ID-DLO, Lelystad, The Netherlands htAFLP, PCR-RFLP

(obtained from M.B. **Skirrow**, PHLS, UK)

NCTC 11168-3 enteritis **NRC**, Ottawa, Canada htAFLP, PCR-RFLP

NCTC 11168-4 enteritis **UMC** Utrecht, The Netherlands htAFLP, PCR-RFLP

NCTC 11168-5 enteritis **LMG** culture collection, Gent, Belgium htAFLP, PCR-RFLP

NCTC 11168-6 enteritis **CCUG** culture collection, Göteborg, Sweden htAFLP, PCR-RFLP

GB1 GBS The Netherlands PCR-RFLP

GB2 GBS The Netherlands PCR-RFLP

GB3 GBS The Netherlands PCR-RFLP

GB4 GBS The Netherlands PCR-RFLP

GB5 GBS The Netherlands PCR-RFLP

MF6 MFS The Netherlands PCR-RFLP

MF7 MFS The Netherlands PCR-RFLP

MF8 MFS The Netherlands PCR-RFLP

GB11 GBS The Netherlands htAFLP, PCR-RFLP

GB13 GBS The Netherlands PCR-RFLP

GB14 GBS The Netherlands PCR-RFLP

GB15 GBS The Netherlands PCR-RFLP

GB16 GBS Belgium PCR-RFLP

GB17 GBS The Netherlands PCR-RFLP

GB18 GBS The Netherlands PCR-RFLP

GB19 GBS The Netherlands PCR-RFLP

MF20 MFS The Netherlands PCR-RFLP

GB21 GBS The Netherlands PCR-RFLP

GB23 GBS The Netherlands PCR-RFLP

GB24 GBS The Netherlands PCR-RFLP

GB25 GBS The Netherlands PCR-RFLP

GB26 GBS The Netherlands PCR-RFLP

GB27 GBS The Netherlands PCR-RFLP

GB29 GBS The Netherlands PCR-RFLP

GB30 GBS The Netherlands PCR-RFLP

E97-0737 enteritis The Netherlands PCR-RFLP

E97-0747 enteritis The Netherlands PCR-RFLP

E97-0796 enteritis The Netherlands PCR-RFLP

E97-0873 enteritis The Netherlands PCR-RFLP

E97-0903 enteritis The Netherlands PCR-RFLP

E97-0921 enteritis The Netherlands PCR-RFLP

E97-0974 enteritis The Netherlands PCR-RFLP

E97-0980 enteritis The Netherlands PCR-RFLP

E97-0998 enteritis The Netherlands PCR-RFLP

E97-1013 enteritis The Netherlands PCR-RFLP

E98-623 enteritis The Netherlands PCR-RFLP

E98-624 enteritis The Netherlands PCR-RFLP

E98-682 enteritis The Netherlands PCR-RFLP

E98-706 enteritis The Netherlands PCR-RFLP

E98-1033 enteritis The Netherlands PCR-RFLP

E98-1087 enteritis The Netherlands PCR-RFLP

cura 7 GBS Curaçao, Netherlands Antilles htAFLP, PCR-RFLP

cura 69 GBS Bonaire, Netherlands Antilles PCR-RFLP

cura 276 GBS Curaçao, Netherlands Antilles htAFLP, PCR-RFLP

260.94 GBS South Africa (HS:41) htAFLP, PCR-RFLP

233.94 GBS South Africa (HS:41) htAFLP

233.95 GBS South Africa (HS:41) htAFLP

308.95 GBS South Africa (HS:41) htAFLP

367.95 GBS South Africa (HS:41) htAFLP

370.95 GBS South Africa (HS:41, same patient as 367.95) htAFLP

242.98 MFS South Africa (HS:41) htAFLP

378.96 enteritis South Africa (HS:41) htAFLP

386.96 enteritis South Africa (HS:41, same patient as 378.96) htAFLP

199.97 enteritis South Africa (HS:41) htAFLP

250.97 enteritis South Africa (HS:41) htAFLP

242.97 enteritis South Africa (HS:41) htAFLP

21.97 enteritis South Africa (HS:41) htAFLP

----------------------------------------------------------------------------------------------------------------------------------------------------------
