# Supplementary material for: Identification of DNA sequence variation in Campylobacter jejuni strains associated with the Guillain-Barré syndrome by high-throughput AFLP analysis
Source: BMC Microbiol. 2006 Apr 4;6:32. doi: 10.1186/1471-2180-6-32 (PMC1513382; doi:10.1186/1471-2180-6-32)
Supplement: Additional file 2 — Primer sequences used in the PCR-RFLP analysis for the validation of potential GBS markers Description of data: (none, title sufficiently describes data) [file 1471-2180-6-32-S2.doc]

## Additional file 2 - Primer sequences used in the PCR-RFLP

## analysis for the validation of potential GBS markers

--------------------------------------------------------------------------------

Marker nr primers

2 5’-CCTGATCATCTTTCTTGGCATGG-3'

5’-AAGATCTACACCCTTATCATCTCC-3'

3 5’-AGAAGTGTATTAACAACCTTGC-3'

5’-ATCATACCGATAATCATCAAAGG-3'

5,6 5’-AGCCACTCAAGCAAATACTAC-3'

5’-AATAAGGAGCACCATTTAAGG-3'

7 5’-AAGATTATTGGCGATAATCC-3'

5’-ATAGATACTATCAGCACTCGC-3'

8 5’-GTTATTTCAAGCATCATAGTCG-3'

5’-ATTTGTCAAAGAATTAGCTCG-3'

9 5’-GCATTAGAAAGTTGCATTAACC-3'

5’-TTCTTCGCAAGCATTAAGTTC-3'

11 5’-GATGGAGCCAAAGAGCTTGTG-3'

5’-CACTTGCAGCAGATAAAGCCG-3'

12 5’-GTCAAAGGCGTTCGGATG-3'

5’-AGCATTGATATGATCAATAGC-3'

13 5’-GCTATTGATCATATCAATGCT-3'

5’-ATCTTCTTTACTATGATAACTCAC-3'

14 5’-GGGTGATATTTCATATCTTGG-3'

5’-GCATAAGCTAAATCCTGTCC-3'

--------------------------------------------------------------------------------
